# A mouse model of the schizophrenia-associated 1q21.1 microdeletion syndrome exhibits altered mesolimbic dopamine transmission

**DOI:** 10.1038/s41398-017-0011-8

**Published:** 2017-11-30

**Authors:** Jacob Nielsen, Kim Fejgin, Florence Sotty, Vibeke Nielsen, Arne Mørk, Claus T. Christoffersen, Leonid Yavich, Jes B. Lauridsen, Dorte Clausen, Peter H. Larsen, Jan Egebjerg, Thomas M. Werge, Pekka Kallunki, Kenneth V. Christensen, Michael Didriksen

**Affiliations:** 1 Division of Synaptic Transmission, H. Lundbeck A/S, Valby, Denmark; 2 Division of Neurodegeneration, H. Lundbeck A/S, Valby, Denmark; 3 Department of Molecular Screening, H. Lundbeck A/S, Valby, Denmark; 4 Invilog Research Ltd and School of Pharmacy, University of Eastern Finland, Kuopio, Finland; 5 Institute of Biological Psychiatry, Mental Health Services of Copenhagen, University of Copenhagen & The Lundbeck Foundation’s IPSYCH Initiative, Copenhagen, Denmark

## Abstract

1q21.1 hemizygous microdeletion is a copy number variant leading to eightfold increased risk of schizophrenia. In order to investigate biological alterations induced by this microdeletion, we generated a novel mouse model (*Df(h1q21)/+)* and characterized it in a broad test battery focusing on schizophrenia-related assays. *Df(h1q21)/+* mice displayed increased hyperactivity in response to amphetamine challenge and increased sensitivity to the disruptive effects of amphetamine and phencyclidine hydrochloride (PCP) on prepulse inhibition. Probing of the direct dopamine (DA) pathway using the DA D1 receptor agonist SKF-81297 revealed no differences in induced locomotor activity compared to wild-type mice, but *Df(h1q21)/+* mice showed increased sensitivity to the DA D2 receptor agonist quinpirole and the D1/D2 agonist apomorphine. Electrophysiological characterization of DA neuron firing in the ventral tegmental area revealed more spontaneously active DA neurons and increased firing variability in *Df(h1q21)/+* mice, and decreased feedback reduction of DA neuron firing in response to amphetamine. In a range of other assays, *Df(h1q21)/+* mice showed no difference from wild-type mice: gross brain morphology and basic functions such as reflexes, ASR, thermal pain sensitivity, and motor performance were unaltered. Similarly, anxiety related measures, baseline prepulse inhibition, and seizure threshold were unaltered. In addition to the central nervous system-related phenotypes, *Df(h1q21)/+* mice exhibited reduced head-to tail length, which is reminiscent of the short stature reported in humans with 1q21.1 deletion. With aspects of both construct and face validity, the *Df(h1q21)/+* model may be used to gain insight into schizophrenia-relevant alterations in dopaminergic transmission.

## Introduction

Schizophrenia symptoms are commonly divided into three domains: positive symptoms (hallucinations, delusions), negative symptoms (deficits in speech, motivation, and social functioning), and cognitive deficits. The arguably most prominent theory of the biology of schizophrenia, the dopamine (DA) hypothesis, is largely based on pharmacological observations: positive symptoms in patients are reduced by DA D2 receptor antagonists, which is the key mechanism of action of all marketed drugs for treatment of schizophrenia^1^. Amphetamine and other compounds that increase DA release increase psychotic symptoms in schizophrenia patients at doses that have no psychotropic effects in healthy volunteers^2^, while higher doses or prolonged exposure can induce psychotic symptoms in healthy volunteers^3^. This has led to the hypothesis that increased DA transmission in the striatum underlies positive symptoms in schizophrenia, and is further supported by imaging data suggesting both increased striatal DA and pre-synaptic DA synthesis capacity in patients with schizophrenia^4,5^. Furthermore, recent genome-wide association studies have shown that single-nucleotide polymorphisms (SNPs) in the D2 receptor are linked to schizophrenia risk^6^. In an extension of this hypothesis, cortical hypodopaminergia has been proposed to underlie positive and negative symptoms, but the evidence is sparser, although recent imaging data lend support^7,8^. While some insight into transmitter alterations has been gained, there is at present no coherent understanding of the etiology that may underlie these changes.

Animal models with construct validity are key tools in the investigation of mechanisms underlying human diseases—and in the search for new drugs. Schizophrenia is a strongly heritable disease^6^, so genetic animal models constitute an obvious tool for investigating the mechanisms underlying this disease. However, until recently only a few reproducible genetic links had been found. In 2008, it became clear that certain rare recurrent hemizygous copy number variants (CNVs) strongly increase the risk of developing schizophrenia^9,10^. Since then, the list has been expanded to at least eight CNVs that increase the risk of developing schizophrenia with genome-wide significance^11,12^. These CNVs provide the arguably best available starting points for the development of genetic animal models of schizophrenia, since their large impact on schizophrenia risk increases the chance of finding relevant phenotypes. Furthermore, unlike, for example, schizophrenia-associated SNPs, the relevant corresponding mouse alterations for CNV models are clear—deletion or duplication of a specific set of genes.

**Fig. 1:**
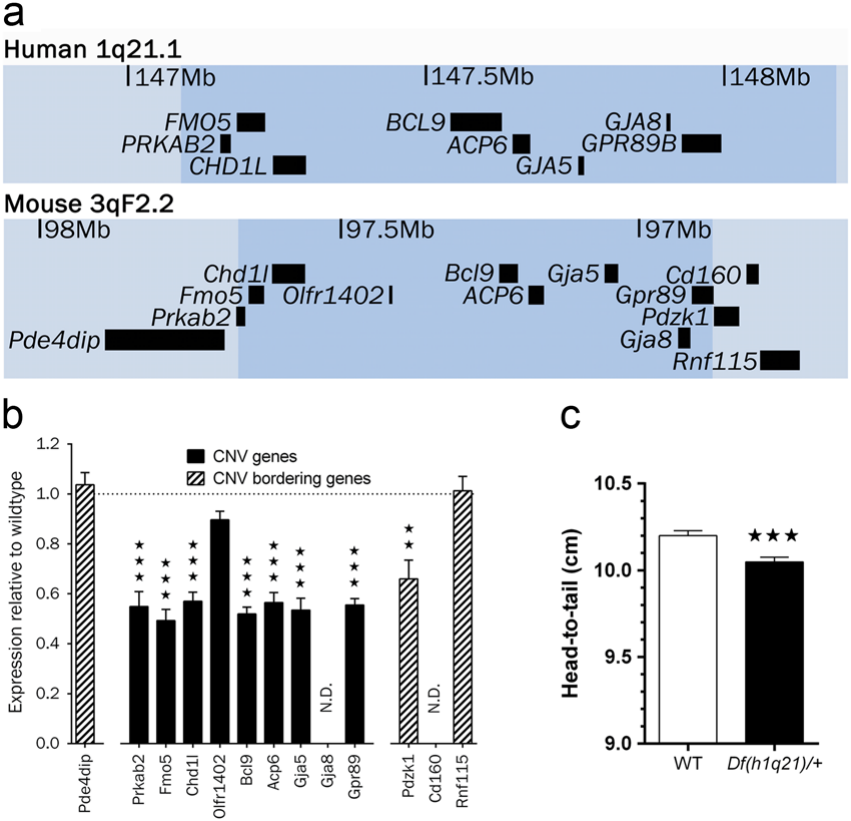
**Construct similarities in** ***Df(h1q21)/+*** **mice and human 1q21.1 deletion carriers. a** Overview of the deleted region in man (1q21.1) and the corresponding orthologous region in mouse (3qF2.2). The mouse deletion spans from a breakpoint upstream of exon 11 of the Gpr89 gene and a breakpoint downstream of exon 7 of the Prkab2 gene (see Supplement 1 for details). The lists are based on the human library GRCh38/hg38 and the mouse library GRCm38/mm10 from the UCSC database. Only annotated RefSeq genes are shown. **b** mRNA expression changes in tissue from frontal cerebral cortex measured by microarray in 14-week-old Df(h1q21)/+ mice compared with their wild-type littermates. The number of genes tested upstream and downstream of the deletion was increased until transcripts could be detected. Gja8 and Cd160 were below the detection limit in wild-type mouse brain. *n* = 6 Df(h1q21)/+ and 12 wild-type littermates. Data presented as median ± SEM. ^***^ *p* < 0.001 following *t*-test with Welch’s correction of gene expression in *Df(h1q21)/+* compared to wild-type mice. **c** Head-to-tail base length. *n* = 233–235 mice/group. Data presented as means ± SEM. ^***^ *p* < 0.001 following Mann–Whitney test. ^**^ *p* < 0.01

The human 1q21.1 microdeletion was found to be associated with eightfold increased risk of schizophrenia in 2008^9,10^, and this association has been replicated in several studies^11,12^. The CNV involves hemizygous deletion of nine genes in the distal part of 1q21.1 (Fig. 1a). In addition to increased risk of schizophrenia, it is also linked to an increased risk of attention deficit hyperactivity disorder (ADHD), developmental delay, and autism spectrum disorders^13,14^. Furthermore, the deletion has been associated with congenital heart defects, facial abnormalities, microcephaly, and short stature^14–16^. Increased risk of additional disorders or syndromic presentation is a shared feature for schizophrenia-associated CNVs, and the risk of additional disorders is also common for SNPs associated with schizophrenia^17,18^. While the 1q21.1 microdeletion strongly increases the risk of schizophrenia and other conditions, some carriers are apparently clinically unaffected by the deletion^14^.

In this paper, we report the generation of the first 1q21.1 microdeletion mouse model *Df(h1q21)/+*, with a functional and pharmacological characterization focusing on schizophrenia-related traits. We find selective alterations in DA transmission and examine how these phenomena depend on pre-synaptic and post-synaptic dopaminergic function. In addition, we find somatic characteristics in the *Df(h1q21)/+* mice that seem to recapitulate clinical observations in human carriers. Overall, these findings suggest that the *Df(h1q21)/+* mouse model constitutes a valuable tool for investigating the functional impact of excessive or dysregulated DA transmission, and putative disease mechanisms underlying positive symptoms of schizophrenia.

## Materials and methods

### Animals

The *Df(h1q21)/+* mouse line was generated by TaconicArtemis (Köln, Germany). Two targeting vectors were generated using bacterial artificial chromosome clones from the C57BL/6J RPCI-23 bacterial artificial chromosome library and transfected into TaconicArtemis C57BL/6N Tac embryonic stem cell line. The first vector introduced a loxP site upstream of the Gpr89 gene. The second vector introduced a loxP site downstream of Prkab2. Homologous recombinant clones were isolated and the 0.8 megabase region on mouse chromosome 3 between the loxP sites was removed by Cre-mediated recombination. Hemizygotic embryonic stem cells were injected into blastocysts isolated from impregnated BALB/c female mice and transferred to pseudopregnant NMRI female mice. Chimeric male pups were selected by coat color and mated with wild-type C57BL/6 female mice. Finally, a chimera with germline transmission was selected for expansion breeding (see Supplemental Methods).

All studies were carried out in accordance with Danish legislation, granted by the animal welfare committee, appointed by the Danish Ministry of Food, Agriculture and Fisheries–Danish Veterinary and Food Administration. Animals were bred by mating wild-type C57BL/6N female mice with hemizygotic *Df(h1q21)/+* male mice to avoid any placental or maternal care effects of the deletion. After weaning at 3 weeks, tail biopsies were collected for polymerase chaine reaction-based genotyping (Supplemental Methods). Mice were then group-housed (two wild-type mice and two hemizygotes from the same litter per cage). Testing was conducted using 9–13-week-old male mice. Animals were randomized into dose groups where appropriate, dependent on genotype. Experimenters were blinded to genotype in all non-automated assays.

### Drugs

Phencyclidine hydrochloride (PCP, Sigma-Aldrich, Denmark), amphetamine (Dexamphetamine, Fagron, UK), apomorphine hydrocloride (Sigma-Aldrich), and quinpirole hydrocloride were all dissolved in 0.9% saline and pH-adjusted where appropriate. SKF-81297 hydrobromide (synthesized at Lundbeck) was dissolved in isotonic water with 5% glucose and pH-adjusted. Compounds were injected subcutaneously at a volume of 10 ml/kg.

### Baseline and psychostimulant-induced activity

For baseline activity, mice were placed individually in Macrolon locomotor activity cages (20 cm × 35 cm × 18 cm, Ellegaard Systems, Denmark) containing an in-chamber activity wheel (Med Associates, USA). Cages were equipped with two rows of 5 × 8 infrared light sources and photocells. Locomotor activity, rearing and running wheel revolutions were recorded for 24 h.

For psychostimulant-induced activity, mice were placed in similar cages with one row of photo beams and no running wheel, and were allowed to habituate for 60 min. Then vehicle, d-amphetamine, or PCP was administered and locomotor activity was recorded for an additional 60 min. To avoid stationary movement artifacts, motility counts required two consecutive crossings of adjacent infrared light beams. Registration and timing of locomotor activity was fully automated (custom-designed hardware and software by Ellegaard Systems).

### Apomorphine-induced climbing

Each mouse was habituated to a small Macrolon cage without bedding for 1 h. Apomorphine was administered 15 min prior the test. Mice were then singly placed in a mesh cylinder (height 14 cm, diameter 13 cm) with a top cover, and time on mesh walls with two or more paws was measured for 120 s.

### Prepulse inhibition

Prepulse inhibition (PPI) testing was performed using the SM100 Startle Monitor System (Kinder Scientific, USA), consisting of eight sound-attenuated startle chambers and Startle Monitor software. Animals were placed in an adjustable Plexiglas holder, providing limited movement but not restraint, positioned directly above a sensing platform. Each test session consisted of 5 min acclimatization with only background white noise (62 dB), followed by habituation with 32 startle pulses of 105 dB (intertrial interval (ITI): 10 s). Animals were then subjected to five types of trials presented 12 times each in a balanced manner: pulse alone, prepulse + pulse (5, 10, or 15 dB above background), or highest prepulse intensity (77 dB) alone. ITI varied from 9 to 21 s (average 15), interstimulus interval was 100 ms, prepulse length 20 ms, and pulse length 30 ms. Each PPI session ended with eight startle pulses of 105 dB to estimate habituation across PPI trials. The full PPI test lasted about 28 min.

PPI was calculated as % PPI for each prepulse intensity as: 100−((prepulse + pulse/pulse_alone) × 100); lower percentage indicates decreased PPI. Startle magnitude was calculated from average of pulse alone trials.

### Ex vivo measurement of DA receptor levels by radio-ligand binding to homogenized striatal tissue

Whole striati were homogenized in 1 ml 0.4 M sucrose and centrifuged at 4 °C for 10 min at 1000×*g*. The supernatant was transferred to a fresh tube and centrifuged at 4 °C for 30 min at 40,000×*g*. The supernatant was discarded and the pellet was re-suspended in 0.5 ml 0.4 M sucrose and protein concentration was measured using a Pierce BCA kit (ThermoFischer, USA).

Binding experiments were performed in a total volume of 200 μl, where 40 μg of homogenate was mixed with either 1 nM ^3^H-SCH-23390 for D1 (PerkinElmer, NET930) or 0.5 nM ^3^H-spiperone for D2 (PerkinElmer, NET565) in binding buffer (50 mM Tris pH 7.4, 120 mM NaCl, 4 mM MgCl_2_, 5 mM KCl, 1 mM EDTA). Nonspecific binding was determined by further addition of 10 μM Flupentixol (for D1) or 10 μM Haloperidol (D2). Binding mixtures were incubated for 30 min at 37 °C before bound ligand was separated by filtration (Packard GF/C unifilter) on a Tomtec Harvester (96® Mach-3u, Tomtec, USA). Filters were washed twice with 0.5 mL ice-cold 50 mM Tris pH 7.7. Filters were dried for 20 min (37 °C) before addition of OptiPhase SuperMix (Perkin Elmer) and counted in a MicroBeta^®^ TriLux (Perkin Elmer) counter for 5 min.

Individual homogenates from six wild-type and six transgenic mice were used to determine D1 and D2 binding in two independent experiments performed in duplicates. Specific binding of *Df(h1q21)/+* mice was normalized to the average specific binding of wild-type mice.

### In vivo voltammetry

Mice were anesthetized by intraperitoneal injection of chloral hydrate (450 mg/kg; Sigma-Aldrich, USA) supplemented by a dose of 100 mg/kg every 45–60 min. Rectal temperature was monitored and maintained at 37 °C using a heating lamp. Animals were fixed in a stereotaxic frame (David Kopf Instruments, USA). Openings were made in the skull and a carbon fiber working electrode (32 µm diameter, 300 µm exposed length) was inserted into the nucleus accumbens (coordinates: 1.42 mm anterior to bregma, 1.0 mm lateral to the midline, and 4.5 mm ventral to the cortical surface)^19^. A bipolar stimulating electrode (diameter 0.35 mm) was inserted into the medial forebrain bundle (MFB; 1.6 mm posterior to bregma, 1.1 mm lateral to the midline, and 5.1–5.3 mm ventral to the cortical surface). The depth of the stimulating electrode was adjusted to evoke maximal DA release. An Ag/AgCl reference electrode was placed on the skull via a saline bridge.

Fast scan cyclic voltammetry was performed and data were analyzed with Invilog Voltammetry Setup (Invilog Research, Finland). The applied potential of a triangular waveform was ramped up from −0.4 to 1.2 V and back to −0.4 V at a scan rate of 300 V/s vs. an Ag/AgCl reference electrode. At the end of the experiment, the location of the working electrode was marked by an electrolytic lesion (6 V, 10 s) for histological visualization.

The MFB was stimulated by 1 ms bipolar 200–300 µA pulses. Frequency–response curves were established for each animal using consecutive bursts of 50 pulses at 10, 20, 30, and 50 Hz applied to the MFB with 1–4 min intervals. Efficiency of DA reuptake was evaluated by measuring the half-width of the descending part of DA overflow peaks of comparable heights (<0.5 μM). Autoinhibition of DA release was assessed by applying paired stimuli (5 bipolar 100 Hz pulses) at variable intervals of 0.5–5 s, and expressed as percentage of the amplitude of DA overflow evoked by the second relative to the first stimulus.

### In vivo microdialysis

Mice were anesthetized with sevoflurane and intracerebral guide cannulas (040, Brainlink, the Netherlands) were stereotaxically implanted into the nucleus accumbens (coordinates: 1.1 mm anterior to bregma, lateral 1.5 mm, and 3.4 mm ventral to dura)^19^. Anchor screws and acrylic cement were used for fixation of the guide cannulas. The body temperature of the animals was monitored by rectal probe and maintained at 37 °C. Mice were allowed to recover from surgery for 1 day, housed individually in cages. On the day of the experiment, a microdialysis probe (Brainlink, PAN 060-10, 1 mm length) was inserted through the guide cannula. Probes were connected via a dual channel swivel to a microinjection pump. Perfusion of the microdialysis probe with filtered Ringer solution (145 mm NaCl, 3 mM KCl, 1 mM MgCl_2_, 1.2 mM CaCl_2_) was begun shortly before insertion of the probe into the brain and continued for the duration of the experiment at a constant flow rate of 1 μl/min. Dialysates were collected every 20 min. After the experiments, brains were removed and probe placement verified. Perfusion for 180 min was followed by collection of four basal fractions and six post-injection fractions. DA in the dialysates was analyzed by HPLC with electrochemical detection, as described previously^20^.

### In vivo electrophysiology

Animals were anesthetized with an intraperitoneal injection of urethane (1.2–1.5 g/kg), mounted in a stereotaxic frame and their temperature was adjusted to 37.5 °C by a heating pad. A hole was drilled above the ventral tegmental area (VTA). Extracellular single-cell recordings were performed using electrodes pulled from glass capillaries and filled with 2% Pontamine Sky Blue in 0.5 M sodium acetate (impedance 2.0–8.0 MΩ at 135 Hz). The electrode was lowered to the dorsal border of the VTA, and then advanced at a slow (1–3 µm/s), uniform speed using a microdrive. Extracellular action potentials were amplified, discriminated, and monitored on an oscilloscope and an audiomonitor.

To assess basal firing properties of DA neurons in the VTA, the number of spontaneously active DA neurons was determined in six to nine stereotaxic descents separated from each other by 150–200 µm. Descents were made in a stereotaxically defined block of tissue within the VTA area (3.4–3.0 mm posterior to bregma, 0.3–0.6 mm lateral to the midline, and 3.8–5.0 mm ventral to the cortical surface)^19^.

Presumed dopaminergic neurons were characterized by a slow and irregular firing pattern (0.5–10 Hz), and triphasic action potentials with a predominant positive component, a negative component followed by a minor positive component, with an overall duration >2.5 ms^21^. The number of spontaneously active DA neurons was counted. Each neuron was recorded for 2–5 min for offline analysis of their basal firing rate, and the coefficient of variation of the interspike interval defined as the ratio between the average interspike interval and the SD of the interspike interval × 100. In addition, the neuronal discharge pattern of each neuron was classified as regular, irregular, or bursty based on autocorrelograms as described previously^22,23^.

The effect of *d*-amphetamine or quinpirole on neuronal firing rate was assessed in a single neuron per animal. Once a DA neuron was isolated and had a stable firing rate for a minimum of 200 s, a single saline injection was performed subcutaneously (5 ml/kg), followed by three consecutive injections of either *d*-amphetamine (1.25, 2.5, and 5 mg/kg) or quinpirole (0.05, 0.10, and 0.20 mg/kg) separated by 15 min. The effect of vehicle and drugs was normalized to the baseline firing rate.

### Data and statistical analysis

Sample sizes were based on established practice in respective assays, and to some extent determined by breeding availability. Statistical analysis was performed using SigmaPlot 11.0 (Systat Software, USA) or Prism 5 (GraphPad Software, USA). Distribution of DA neuron firing patterns was and using the *Χ* ^2^-test. All other data were analyzed by either two-way analysis of variance (ANOVA), two-way mixed-model ANOVA, or by *t*-test. Where appropriate, *t*-test was replaced with Mann–Whitney rank sum test. Post hoc tests following ANOVAs were conducted using Bonferroni correction. Two-tailed levels of significance were used and *p* < 0.05 was considered statistically significant.

## Results

### Generation of *Df(h1q21.1)/+* mice and expression of targeted genes

Mouse orthologs of the human 1q21.1 genes are located on mouse chromosome 3F2.2 (Fig. 1a). A mouse model of the human 1q21.1 microdeletion syndrome (*Df(h1q21.1)/+*) was generated by deleting the corresponding region on mouse chromosome 3qF2.2 through Cre-mediated recombination. Pups were born at the expected ratios (hemizygous fraction 0.49, 95% confidence interval: 0.41–0.56, *n* = 179).

Expression of 1q21.1 genes was examined by microarray analysis of RNA from the cerebral cortex (Fig. 1b). Except for the olfactory receptor gene *Olfr1402*, all detected genes in the deleted region showed significant downregulation to roughly 50% of the expression in wild-type mice. Cortical RNAseq data from wild-type mice did not detect any Olfr1402 transcript, suggesting that the apparently unchanged Olfr1402 expression reflects false detection in the microarray experiment (data not shown). Expression of one flanking gene, *Pdzk1*, was also reduced by 50% in *Df(h1q21)/+* mice.

### *Df(h1q21.1)/+* mice are shorter than wild-type mice similar to human deletion carriers

Short stature has been reported as a common phenotype of the human 1q21.1 microdeletion^13,14^, and we therefore measured the length of adult *Df(h1q21)/+* mice. Like the reported short stature of human carriers, *Df(h1q21)/+* mice were shorter than wild-type littermates (*p < *0.001, Fig. 1c).

Adult *Df(h1q21)/+* mice had grossly normal behavior and appearance, but were slightly lighter than wild-type littermates (wild-type 24.2 ± 0.1 g, *n* = 233; *Df(h1q21)/+*23.0 ± 0.1 g, *n* = 235; *t*-test, *p* < 0.0001). We found no alterations in gross brain morphology, myelination, and parvalbumin-positive interneuron counts (Supplementary Fig. S1).

### Df(h1q21)/+ mice exhibit increased sensitivity to psychostimulants

*Df(h1q21)/+* mice were examined in a broad behavioral test battery focusing on schizophrenia-related traits, but also including measures of motor performance, pain sensitivity, anxiety, and seizure threshold (see Supplementary Table S1 for an overview of assays and results). The *Df(h1q21)/+* mice were indistinguishable from wild-type littermates in most assays in this test battery with two exceptions: slightly reduced activity in 24-h running wheel (Supplementary Fig. S2a) and altered psychostimulant response as detailed below.

**Fig. 2:**
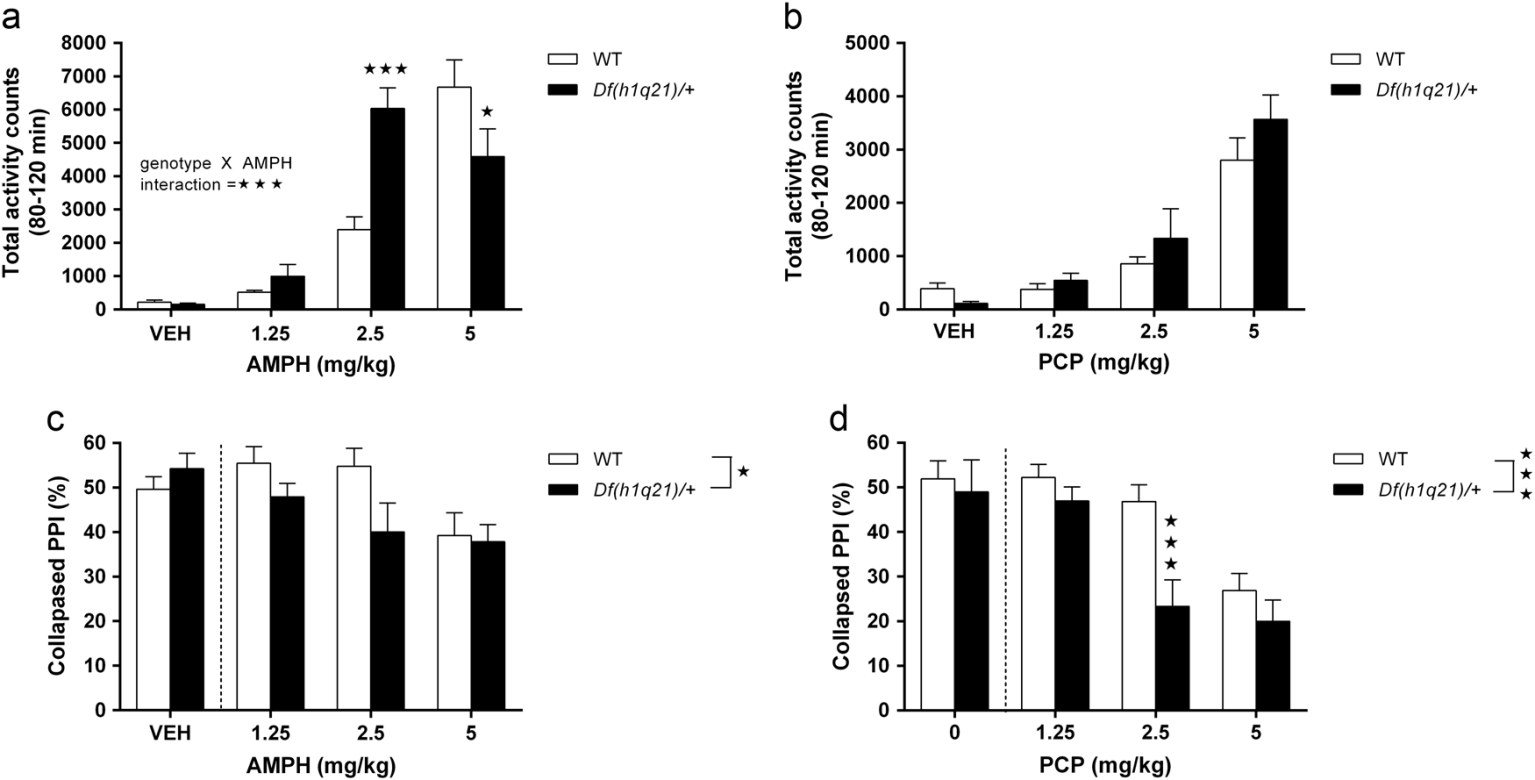
**Psychostimulant-induced behavioral alterations in** ***Df(h1q21)/+*** **mice. a** Horizontal activity following amphetamine (AMPH) treatment (0–5 mg/kg). Genotype × treatment interaction, F_3,88_ = 11.15, *p* < 0.001, *n* = 11–12/group. For full time curve see Supplementary Fig. S3A. **b** Horizontal activity following phencyclidine (PCP) treatment (0–5 mg/kg). *n* = 12/group. No significant differences between genotypes. For full time curve see Supplementary Fig. S3B. **c** Prepulse inhibition (PPI) following AMPH treatment. Effect of genotype, *F*_1,64_ = 4.50, *p* < 0.05, *n* = 11–12/group. **d** Prepulse inhibition (PPI) following PCP treatment. Effect of genotype, *F*_1,61_ = 12.05, *n* = 10–12/group. Data sets were analyzed by two-way ANOVA with post hoc comparisons using Bonferroni correction where appropriate. Stars in the upper right corner of figures represent main effect of genotype, while stars above columns represent Bonferroni-corrected post hoc tests. Data presented as means ± SEM. ^*^ *p* < 0.05; ^***^ *p < *0.001 vs. wild types. Dotted lines in **c**, **d** symbolize exclusion of vehicle-treated animals from statistical analyses (see Results section)

Following amphetamine administration, the locomotor response of *Df(h1q21)/+* mice was different from that of wild-type littermates (*p < *0.001, Fig. 2a). *Df(h1q21)/+* mice showed an increased response to 2.5 mg/kg of amphetamine (*p < *0.001) and a decreased activity at 5 mg/kg amphetamine (*p < *0.05), likely due to increased stereotypy at this dose. The increased locomotor response to 2.5 mg/kg amphetamine was subsequently confirmed in an independent experiment (Supplementary Figs. S2b, c).

Following PCP administration, the locomotor response of *Df(h1q21)/+* mice was not significantly different from wild types (Fig. 2b). We also assessed basal and psychostimulant-disrupted acoustic startle response (ASR) and PPI. Basal ASR at various sound intensities (95–120 dB) was unaltered in *Df(h1q21)/+* mice (Supplementary Fig. S3a). Similarly, since basal PPI was not affected by genotype (Supplementary Fig. S3b), and no genotype × prepulse intensity interaction was observed, only drug-treated groups were included in the analyses of the following psychostimulant experiments, and PPI was collapsed across prepulse intensities.

Amphetamine treatment decreased PPI more in *Df(h1q21)/+* than in wild-type mice (*p < *0.05, Fig. 2c), indicating an increased sensitivity to the disruptive effect of amphetamine on PPI as well. The increased PPI sensitivity to 2.5 mg/kg amphetamine was subsequently replicated in a separate batch of animals (Supplementary Fig. S4c). Amphetamine decreased ASR to a similar extent in both genotypes (Supplementary Fig. S4f). In contrast to the locomotor assay, *Df(h1q21)/+* mice were significantly more sensitive to PCP-induced disruption of PPI than wild types (*p < *0.001, Fig. 2d), which was driven by the 2.5 mg/kg PCP dose (*p < *0.001, Fig. 2d). The effect of PCP on ASR was not different between the two genotypes (data not shown).

Because of the increased amphetamine sensitivity found in the *Df(h1q21)/+* mice, we also measured the brain and blood exposure of amphetamine in the mice 1 h after administration. There were no differences in the brain or plasma levels of amphetamine between genotypes (Supplementary Fig. S6).

### Sensitivity of *Df(h1q21)/+* mice to D1 and D2 receptor agonists and expression of DA receptors

The increased amphetamine sensitivity of *Df(h1q21)/+* mice suggests altered dopaminergic signaling in the basal ganglia, and we therefore investigated post-synaptic and pre-synaptic functions in more detail.

**Fig. 3:**
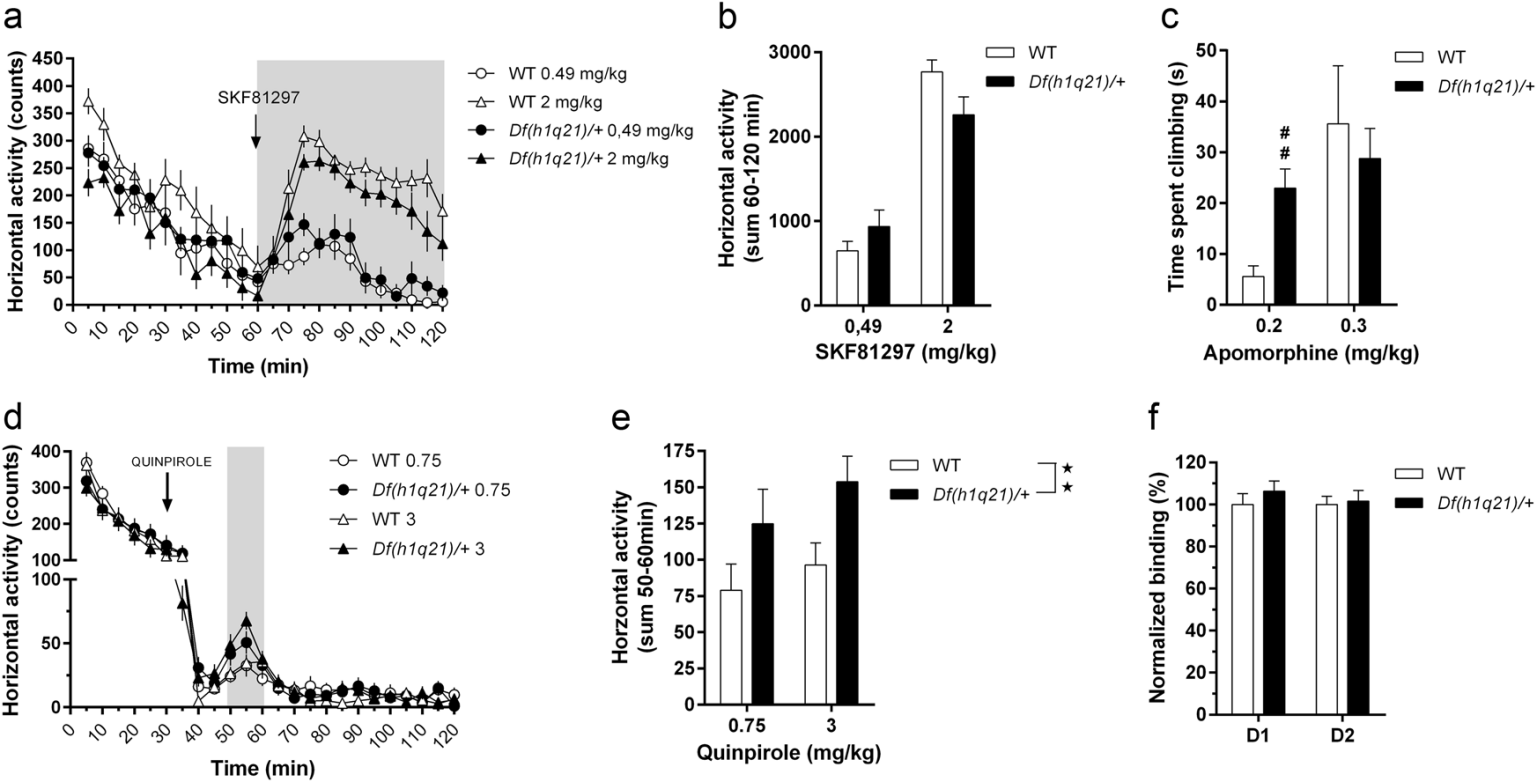
**D1-dependent and D2-dependent changes in** ***Df(h1q21)/+*** **mice. a** Horizontal activity (in 5 min bins) following treatment with D1 agonist SKF-81297 (0.49 and 2 mg/kg). *n* = 7–8/group. Shaded area representing bins used for quantification. **b** Horizontal activity summary for 60–120 bins in (**a**). **c** Climbing duration following two doses of apomorphine (0.2 and 0.3 mg/kg). ^##^ *p < *0.01 following Mann–Whitney test with Bonferroni correction. **d** Horizontal activity (in 5 min bins) following treatment with D2 agonist quinpirole (0.75 and 3 mg/kg). *n* = 7–8/group. **e** Horizontal activity summary for 50–60 bins in (**d**; effect of genotype following mixed model ANOVA). **f** Normalized D1-specific and D2-specific binding using striatal membranes. *n* = 11–12/group. Stars above columns represent post hoc tests and stars in the upper right corner of figures represent main effect of genotype. Data presented as means ± SEM. ^**^ *p < *0.01

First, we assessed potential post-synaptic effects of the deletion using DA receptor agonists. The locomotor response to the D1 agonist SKF-81297 was similar in *Df(h1q21)/+* and wild-type mice (Fig. 3a, b). D2 signaling was then probed by administering the D2/D3 agonist quinpirole and assessing its effects on horizontal activity. Initially, quinpirole strongly decreased activity similarly in both genotypes, presumably because of activation of pre-synaptic autoreceptors (Fig. 3d). As expected, a putatively post-synaptic D2-dependent increase in locomotor activity was seen around 30 min after quinpirole administration (Fig. 3d). This late increase in activity has been hypothesized to primarily reflect post-synaptic D2 activation^24^, and this increase was significantly bigger in *Df(h1q21)/+* mice compared to wild types (*p < *0.01, Fig. 3e). Similarly, administration of the non-selective DA agonist apomorphine caused a significantly higher degree of climbing behavior in *Df(h1q21)/+* mice at 0.2 mg/kg (*p* < 0.01, Fig. 3c) but not at 0.3 mg/kg.

To investigate whether DA receptor levels were altered, we measured binding of tritiated D1 and D2 receptor ligands to striatal membranes from these mice ex vivo (Fig. 3f). There was no difference in D1 and D2 receptor levels between the genotypes, indicating that changes in receptor expression level are unlikely to explain the differences in behavioral response.

### Stimulated DA release, reuptake, and autoinhibition in the nucleus accumbens of *Df(h1q21)/+* mice

Putative pre-synaptic alterations in DA release and reuptake were assessed by in vivo voltammetry.

**Fig. 4:**
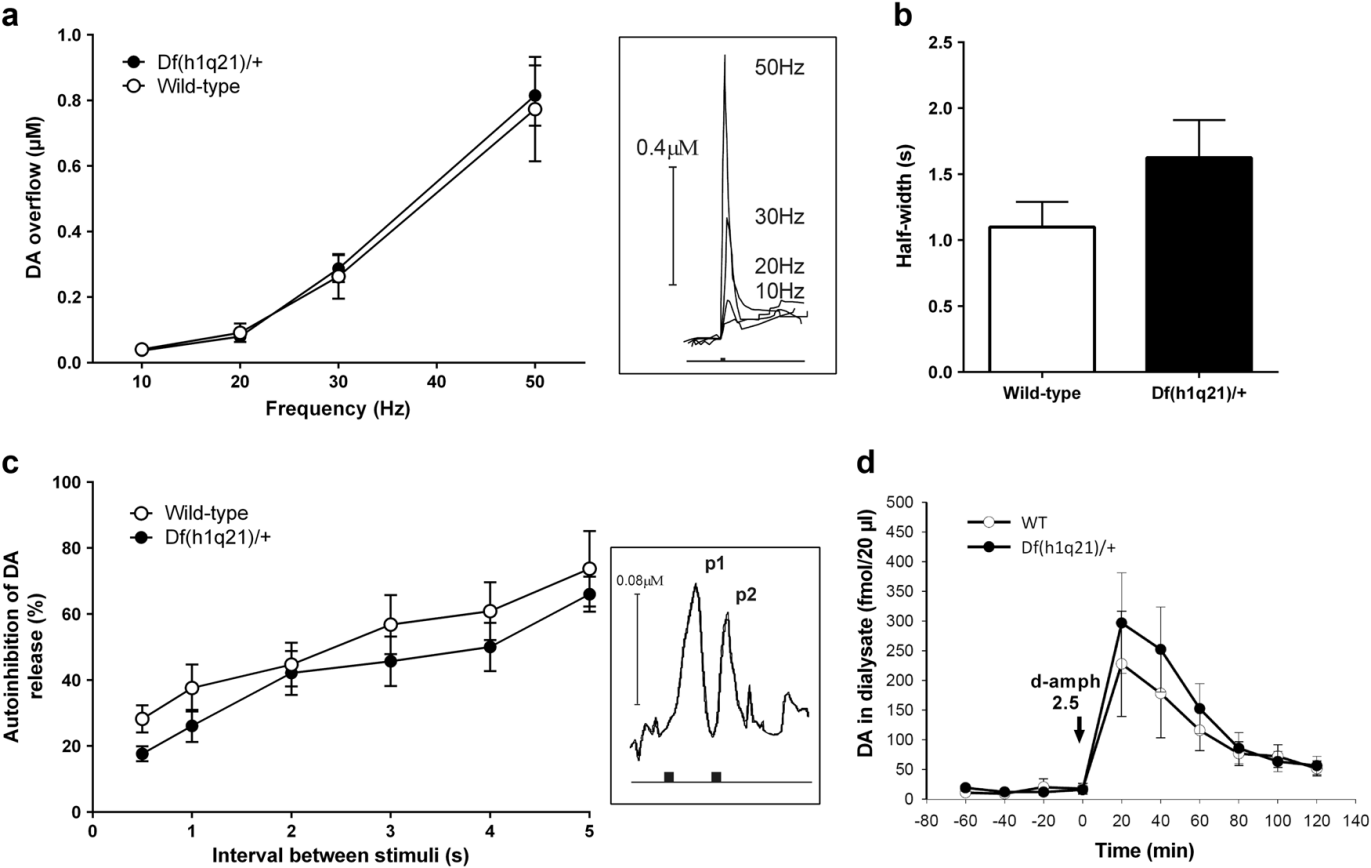
**Dopamine (DA) homeostasis in the nucleus accumbens of** ***Df(h1q21)/+*** **and wild-type (WT) mice. a** DA overflow (µM), determined by voltammetry, in response to increased frequency of MFB stimulation (10–50 Hz) was not different between genotypes (two-way ANOVA with repeated measurements, F_(1,42)_ = 0.43, *p* = 0.53, *n* = 8–9). Insert: representative examples of DA overflow in response to four different stimulation frequencies. **b** DA reuptake was evaluated by measuring the half-width of descending part of the overflow curves at low frequencies of stimulation (10–30 Hz), and was not significantly different between genotypes (unpaired *t*-test, *p* = 0.16, *n* = 7–8). **c** Autoinhibition of DA release was evaluated by application of two consecutive stimuli (interval 0.5 – 5.0 s), and was not significantly different between genotypes (two-way ANOVA with repeated measurements, F_(1,75)_ = 2.64, *p* = 0.13, *n* = 7–8); the amplitude of DA overflow evoked by the second stimulation (p2, insert) was expressed as percentage of the response evoked by the first stimulation (p1, insert). **d** The effect of d-amphetamine (2.5 mg/kg, s.c.) on DA efflux in the nucleus accumbens (fmol/20 µl) was investigated in both genotypes using in vivo microdialysis (two-way ANOVA with repeated measurements, F_(1,60)_ = 0.43, *p* = 0.52, *n* = 6–11)

Electrical stimulation of the MFB using a burst of 50 pulses increased extracellular DA concentrations in the nucleus accumbens in a frequency-dependent, nonlinear manner. This nonlinear relationship between frequency of stimulation and peak DA overflow is suggested to result from a shift of the balance between release and reuptake mechanisms toward dominance of release at higher stimulation frequency^25^, and it was not altered in *Df(h1q21)/+* mice (*p* = 0.53, Fig. 4a).

Efficiency of DA reuptake was assessed by measuring the half-width of the descending slope of the peak DA overflow evoked at low frequencies of stimulation where uptake mechanisms are not saturated (peak DA concentrations below 0.5 µM)^26^. It was not significantly different in Df(h1q21)/+ and wild-type mice (*p* = 0.16, Fig. 4b).

Autoinhibition of DA release mediated by pre-synaptic D2 autoreceptors was evaluated by a paired-pulse stimulation protocol^27^. The interval between the two stimuli was negatively correlated to the autoinhibition, i.e., the shorter the interval, the larger the inhibition of the second DA overflow peak (Fig. 4c). Autoinhibition of DA release was not significantly different in Df(h1q21)/+ mice compared to wild-type mice (*p* = 0.13, Fig. 4c).

### d-amphetamine-induced DA efflux in the nucleus accumbens of *Df(h1q21)/+* mice

Extracellular DA levels in the nucleus accumbens were assessed by microdialysis. Basal DA levels without considering probe recovery were 7.7 ± 2.2 in wild type (*n* = 6) and 12.2 ± 3.8 (*n* = 11) fmol/20 µl in *Df(h1q21)/+*, and were not significantly different between genotypes (*p* = 0.96).

Injection of *d*-amphetamine induced DA efflux reaching a maximum 20 min following administration in both wild-type and *Df(h1q21)/+* mice (Fig. 4d). There was no significant difference between genotypes (*p* = 0.52).

### Basal firing properties of DA neurons in the ventral tegmental area of *Df(h1q21)/+* mice

**Fig. 5:**
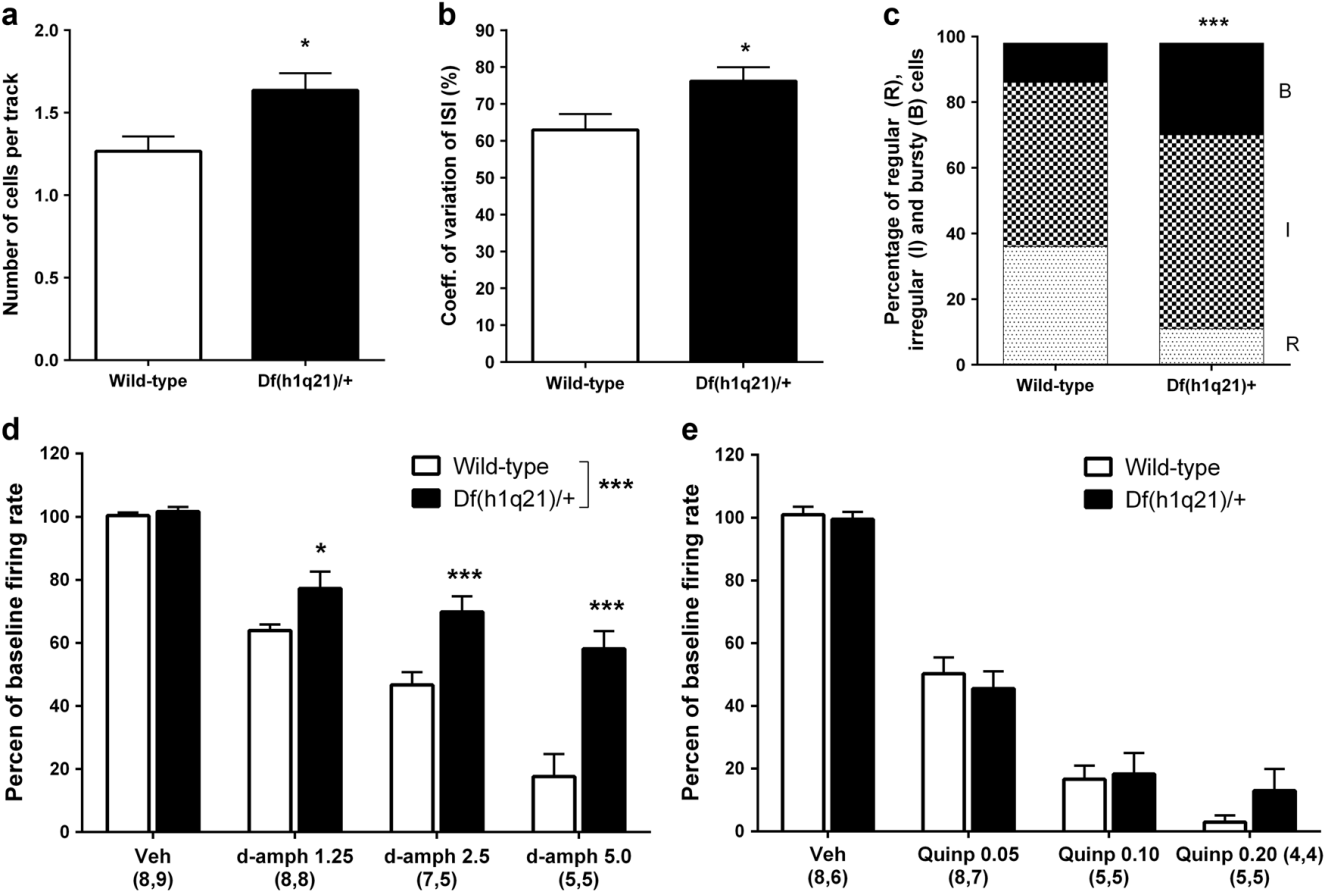
**Electrophysiological properties of dopaminergic neurons in the VTA of** ***Df(h1q21)/+*** **and wild-type (WT) mice. a** The number of spontaneously active DA cells per track was significantly higher in Df(h1q21)/+ compared to wild-type mice (unpaired *t*-test, *p* = 0.028, *n* = 57–84). **b** Spike trains for all active neurons in each genotype were analyzed to calculate the coefficient of variation of the interspike interval (ISI), which was significantly higher in *Df(h1q21)/+* compared to wild-type mice (unpaired *t*-test, *p* = 0.012, *n* = 57–84). **c** The firing pattern was classified as regular, irregular, or bursty for each neuron recorded, and a significantly different distribution was present in Df(h1q21)/+ compared to wild-type mice (*Χ* ^2^-test, *p* < 0.001, *n* = 57–84). **d** The effect of d-amphetamine on DA cell firing rate was evaluated by administration of increasing doses of d-amphetamine (1.25, 2.5, and 5 mg/kg i.v.) following a single vehicle injection (NaCl 0.9%, i.v.); a significant difference in the dose-dependent inhibition was observed between genotypes (two-way ANOVA with repeated measurements, F_(1,32)_ = 27,31, *p* < 0.001, *n* = 5–9). **e** The effect of quinpirole on DA cell firing rate was evaluated by administration of increasing doses of quinpirole (0.05, 0.10, and 0.20 mg/kg i.v.) following a single vehicle injection (NaCl 0.9%, i.v.); no significant difference in the dose-dependent inhibition was found between genotype (two-way ANOVA with repeated measurements, F_(1,23)_ = 0.159, *p* = 0.69, *n* = 4–8). *p < 0.05; ***p < 0.001

Firing properties of DA neurons were assessed in the VTA of *Df(h1q21)/+* (six mice, 84 neurons) and wild-type (five mice, 57 neurons) mice. The average number of spontaneously active DA neurons per track was increased in *Df(h1q21)/+* mice (*p* = 0.028, Fig. 5a). The average firing rate of all recorded DA neurons was not significantly different in *Df(h1q21)/+* mice (3.64 ± 0.30 Hz) compared to wild-type mice (4.04 ± 0.32 Hz; *p* = 0.18, data not shown). The coefficient of variation of the interspike interval was significantly higher in *Df(h1q21)/+* mice compared to wild-type mice (*p* = 0.012, Fig. 5b). Further classification of the firing pattern into regular, irregular, or bursty, for all recorded neurons showed that *Df(h1q21)/+* mice exhibited a significantly different distribution of the different firing patterns compared to wild-type mice (*p* < 0.001, Fig. 5c), with a higher proportion of bursty neurons.

### d-amphetamine and quinpirole-induced inhibition of DA firing activity in *Df(h1q21)/+* mice

Administration of *d-*amphetamine to wild-type mice induced a dose-dependent inhibition of DA cell firing rate in the VTA compared to vehicle administration (*p* < 0.001). Although dose-dependent inhibition was also observed in *Df(h1q21)/+* mice, the suppressive effect of *d-*amphetamine was reduced compared to wild-type mice (*p* < 0.001, Fig. 5d).

Administration of quinpirole to wild-type mice induced a dose-dependent inhibition of DA cell firing rate in the VTA compared to vehicle administration (*p* < 0.001), which was not significantly altered in Df(h1q21)/+ mice (*p* = 0.72).

## Discussion

In this paper, we describe the generation and characterization of the first mouse model of the schizophrenia-associated 1q21.1 CNV. *Df(h1q21)/+* mice exhibited increased response to psychostimulants and altered DA transmission. These changes were relatively specific, as the mice were otherwise indistinguishable from wild-type mice in a broad panel of behavioral tests. The *Df(h1q21)/+* phenotype recapitulates key aspects of the DA hypothesis of schizophrenia, making it a promising model for investigation of schizophrenia-related alterations of DA transmission.

In addition to the altered brain function, the *Df(h1q21)/+* mice exhibited reduced head-to-tail length. Short stature is observed in human subjects with 1q21.1^13,14^, further supporting the translational potential of the model.

### DA phenotype

*Df(h1q21)/+* mice were hypersensitive to the DA-releasing drug amphetamine in locomotor as well as PPI experiments. In addition, they were hypersensitive to the NMDA receptor antagonist PCP in the PPI test. Since altered psychostimulant sensitivity and DA metabolism are among the best validated observations in schizophrenia, these changes support the schizophrenia relevance of the *Df(h1q21)/+* model—particularly with respect to positive symptoms, which are most convincingly linked to DA transmission^1^. Thus, the *Df(h1q21)/+* mice provide an opportunity to study changes in DA transmission in a genetically relevant schizophrenia model.

Further examination of post-synaptic DA function revealed that striatal DA receptor levels and D1 signaling were unaffected in Df(h1q21)/+ mice, while their sensitivity to D2 agonists and mixed D2/D1 agonists was increased. This might indicate that post-synaptic D2 signaling is altered, although the effect may also be mediated by pre-synaptic D2 autoreceptors.

Evaluation of pre-synaptic DA function based on DA levels in the nucleus accumbens and *in vivo* voltammetry indicated that release and reuptake capacity were unaltered in *Df(h1q21)/+* mice. In contrast, DA neuron firing in the VTA was altered in *Df(h1q21)/+* mice with more spontaneously active cells and increased firing variability. Furthermore, attenuated inhibition of firing in response to amphetamine was seen in *Df(h1q21)/+* mice—amphetamine releases DA by firing-independent mechanisms leading to inhibition of DA neuron firing through somatodendritic D2 autoreceptors and accumbal feedback pathways^28^. Our findings therefore indicate altered feedback regulation of DA cell firing in *Df(h1q21)/+* mice compared to wild types. Changes in D2 autoreceptor function are unlikely, since the D2 agonist quinpirole inhibited DA cell firing equally in both genotypes, a mechanism known to involve somatodendritic autoreceptors^29^. Furthermore, autoreceptors located on dopaminergic terminals in the nucleus accumbens were not functionally different in *Df(h1q21)/+* mice compared to wild types as shown by the comparable paired-pulse depression of DA release. Alternatively, reduced feedback regulation of DA neuron firing may increase the number of spontaneously active dopaminergic neurons through reduced GABAergic tone in the VTA^21,30^. This could in turn increase their sensitivity to excitatory inputs and exacerbate burst firing, as observed in the present study. However, the increased population activity and burst firing did not result in a significant increase in extracellular DA in nucleus accumbens. Similar apparent discrepancies have been found in other studies^30,31^. Possible explanations include different sensitivity of dopaminergic systems in anesthetized vs. awake^32,33^, and a minor effect of burst firing on tonic DA^30^. Furthermore, DA levels that are too small to be robustly detected by microdialysis may contribute to the behavioral phenotypes, and compensatory changes occluding an effect on DA release might occur. We focused on the mesolimbic pathway, but meta-analysis of human imaging data suggests that pre-synaptic hyperdopaminergia in schizophrenia is more prominent in associative striatum^8^. Conceivably, dopaminergic alterations in Df(h1q21)/+ mice could be more prominent there, and dopaminergic signaling in associative striatum should be tested in future studies.

In summary, the altered DA cell firing activity clearly supports a pre-synaptic DA dysfunction in *Df(h1q21)/+* mice, but other observations such as unchanged extracellular DA levels and the behavioral effect of a D2 agonist suggest that there are other, possibly compensatory, changes in the *Df(h1q21)/+* DA system. Further studies, including investigations of more dorsal parts of the striatum, are needed to expand the understanding of mechanisms underlying the altered psychostimulant response and DA transmission in *Df(h1q21)/+* mice.

### Comparison to other animal models of schizophrenia

Psychostimulant sensitivity has been examined for two other mouse models of schizophrenia-associated CNVs: the 15q13.3 and the 22q11.2 microdeletions^34–36^. While these two models had specific phenotypes that might relate to schizophrenia, neither of them had altered amphetamine sensitivity, but the 22q11.2 model had increased sensitivity to PCP similar to *Df(h1q21)/+* mice and slightly altered DA metabolism in the cortex^34,36^. This suggests that these chromosomal deletions provoke a complex pattern of partly overlapping, partly distinct neurophysiological perturbations that predispose to severe mental illness. Arguably, this is not surprising, given the partial penetrance of such deletions in humans. Furthermore, there are translational challenges including species differences in brain biology and behavior, suppressant effects of genetic background and age-dependent appearance of phenotypes^37^. Thus, the models may be reliable models of the human CNVs but cannot be considered to be genuine schizophrenia or disease models, but rather as complementary models of liability to mental disorders. It may be possible to strengthen penetrance in models by introducing second hits, e.g., by testing CNV models in different strains that may be more susceptible or combining a CNV model with an environmental challenge. We only profiled male mice to reduce variability and increase power. Female mice should also be examined in future studies.

Several of the so-called neurodevelopmental schizophrenia models based on developmental challenge have altered psychostimulant response^38^. The methylazoxymethanol (MAM) model, where rats are exposed to MAM in utero at embryonic day 17, is the best characterized of those with regard to DA transmission^39,40^. Like the *Df(h1q21)/+* mice, MAM rats have increased locomotor response to amphetamine and VTA population activity at baseline^41,42^. However, no change in burst firing was reported in the MAM model. Hyperactivity of the ventral subiculum has been suggested to cause a hyperdopaminergic state in MAM rats^41,42^, and it would be relevant to investigate whether similar changes occur in *Df(h1q21)/+* mice.

### Gene contributions

Nine genes are hemizygously deleted in the 1q21.1 microdeletion (Fig. 1). Our characterization of *Df(h1q21)/+* mice allows investigation of the contribution of the individual genes to altered DA transmission by comparison with mice with hemizygous deletion of individual genes. Mice with homozygous deletion of the *Prkab2*, *Fmo5*, *Gja5*, and *Gja8* genes have been reported^43–46^. However, none of these studies have tested amphetamine sensitivity of mice with homozygous or hemizygous deletions, leaving this comparison to future work.

### Perspectives

In conclusion, we have generated and characterized the first mouse model of the schizophrenia-associated human 1q21.1 microdeletion syndrome, which we show have altered DA signaling and psychostimulant sensitivity as also observed in patients with schizophrenia. The limited similarity of available genetic schizophrenia models highlights that many paths may lead to schizophrenia, and further the need to characterize and compare different models to understand the convergence to the symptoms of schizophrenia. In this context, an improved description of the schizophrenia symptomatology associated with the individual genetic variants would be of great importance—e.g., whether schizophrenia patients with 1q21.1 microdeletion predominantly display positive symptoms. The perturbations of *Df(h1q21)/+* mice demonstrated here appear to primarily affect the DA system, and particularly D2 receptor-mediated transmission. As this receptor serves as a common target for all existing antipsychotics, the *Df(h1q21)/+* model may prove to be particularly useful for the investigation of mechanisms underlying positive symptoms and the associated aberrant DA transmission observed in schizophrenia.

## Electronic supplementary material

Supplemental Material
