# Supplementary material for: A mouse model of the schizophrenia-associated 1q21.1 microdeletion syndrome exhibits altered mesolimbic dopamine transmission
Source: Transl Psychiatry. 2017 Nov 30;7:1261. doi: 10.1038/s41398-017-0011-8 (PMC5802512; doi:10.1038/s41398-017-0011-8)
Supplement: Supplementary file 1 — Supplemental Material [file 41398_2017_11_MOESM1_ESM.pdf]

Mouse model of 1q21.1 microdeletion syndrome
Supplementary Information

Supplemental Methods

Generation of the Df(h1q21)/+ mouse

The two targeting vectors were constructed as shown in Supplementary Figure 7. The first vector introduced a loxP site with an FRT-flanked Neomycin resistance cassette upstream of exon 11 in the Gpr89 gene, replacing exons 12-14 in the gene. The second vector introduced a loxP site with a F3-flanked Puromycin resistance cassette downstream of exon 7 in the Prkab2 gene, replacing exon 8 in the gene. Both constructs also contained a thymidine kinase gene for negative selection. Targeted sequences were generated using BAC clones from the C57BL/6J RPCI-23 BAC library.

First, linearized Gpr89-targeting vectors were electroporated into the TaconicArtemis C57BL/6N Tac embryonic stem cell (ESC) line. Homologous recombinant clones were isolated by positive (G418 resistance) and negative (Gancyclovir resistance) selection. The genetic construct was further validated using southern blotting to confirm a single integration on only one chromosome.

Secondly, selected ESC lines were electroporated with linearized Prkab2 targeting vector. Homologous recombinant clones were isolated using positive (puromycin resistance) and negative (Gancyclovir resistance) selection. Again, the genetic construct was further validated using southern blotting to confirm a single integration on only one chromosome.

Thirdly, double targeted ESC lines were electroporated with a cre-recombinase expressing construct to facilitate in vitro Cre-mediated recombination in ESC clones with the two loxP sites inserted on the same chromosome. The ESCs hemizygotic for the 800 kb deletion were identified by southern blotting and PCR analysis. PCR analysis was done using primers that span the deletion site – a forward primer fP1 (5'-TAAAGCCGCGGGTCGACTCGA-3') and a reverse primer rP3 (5'-AGAGGCAGTGGATCCTACAAGAGAC-3') – which produce a 114 bp product from the recombined allele.

Finally, selected ESCs were microinjected into blastocysts isolated from impregnated BALB/c females and transferred to pseudopregnant NMRI females. Chimeric male pups were selected by coat color and mated with wildtype C57BL/6 females and a chimera with germline transmission was selected for expansion breeding. Genotypes were controlled during breeding by PCR on tail biopsies.

Brain Anatomy and Immunohistochemistry

For immunohistochemical evaluation, animals were perfusion-fixed with 4% paraformaldehyde. Brains were post-fixed for 4 hours before being embedded in gelatine. Tissue sections were cut at 30 µm for and every 100 µm sections through the entire brain were collected (Neuroscience Associates). The brain sections were incubated with parvalbumin antibody (P3088, Sigma) at 4°C overnight followed by incubation with a secondary biotinylated antibody (Dako) for 1 hour at room temperature. Following secondary antibody the sections were incubated with streptavidin-biotin complex (Vectastain) for 1 hour before developing with diaminobenzidine and mounting with Pertex (HistoLab). Hemotoxylin/eosin and solochrome staining were also performed to observe overall brain structure and myelination patterns. Images were obtained using a Nikon Eclipse E1000 microscope and Image pro plus imaging software.

Basal acoustic startle response (ASR)

Basal ASR was assessed in the same system as PPI (see methods). Briefly, following a 5 min acclimatization time with only background noise (white noise, 62 dB), animals were subjected to 6 different types of startle trials using different intensities (95, 100,105, 110, 115 and 120 dB with a duration of 30ms). Each trial type was presented 12 times in a balanced pseudo-randomized manner. Intertrial intervals varied between 9 and 21 s (average ITI 15 s). The full test lasted approximately 30 min.

Amphetamine exposure

Plasma and brains from mice used in the PPI experiments was collected 1h after administration of amphetamine (s.c., n=6-8/dose). Concentrations of amphetamine in plasma and brain homogenate were measured by means of ultra-performance liquid chromatography coupled with tandem mass spectrometry detection. Separation was achieved using a Waters Acquity UPLC BEH Phenyl column (1.7 µm, 30 mm, 2.1 mm particles) with a mobile phase consisting of ammonium hydroxide in a water-acetonitrile mixture. Amphetamine was detected in positive ion mode with a mass to charge ratio of 136.1 to 91.2 m/z.

Supplemental Results

Table S1. Overview of behavioral profiling package.

Domain	Assay	Finding	Comment
**Basal**	*Viability*	(↓)	Litter size normal at birth, slightly fewer *Df(h1q21)/+* mice at weaning
	Body weight and length	↓	Body length measured from head to tail root
	Irwin test	↔	
**Motor**	*Rota-rod*	↔	
	Beam-walking	↔	
**Pain**	*Thermal hot-plate*	↔	
**Light/Dark Cycle**	*Diurnal activity*	↓	Effect seen in both light and dark phase
**Anxiety**	*Elevated plus maze*	↔	
	Bright open field	↔	
**Seizures**	*MEST*	↔	Trend to increased seizure threshold)
	PTZ seizures	↔	Trend to increased clonic-tonic seizure threshold
**Sensorimotor processing**	*Prepulse inhibition*	↔<br>↓<br>↓	Basal<br>After AMPH challenge (1.25-5 mg/kg)<br>After PCP challenge (1.25-5 mg/kg)
	Acoustic startle response	↔	Basal (95-120dB)
	Acoustic startle response	↔	During PPI test and after AMPH and PCP
**Positive symptoms / Dopamine system**	*Basal motility*	↓	Decreased activity during exploration
	PCP hyperactivity	↔	1.25 -5 mg/kg
	AMPH hyperactivity	↑	1.25 -5 mg/kg
	D1 hyperactivity	↔	SKF81297 (0.49 and 2 mg/kg)
	D2 hyperactivity	↑	Quinpirole (0.75 and 3 mg/kg)
	Apomorphine climbing	↑	At 0.2 mg/kg but not 0.3 mg/kg
**Cognition**	*Spontaneous alternation*	↔	Y-maze
	Fear conditioning (cue and context)	↔<br>↔	Cued memory tested after 5h delay<br>Context memory after 1h delay
	Morris watermaze	↔ (acquisition),<br>↔ (24h probe)	4 days acquisition<br>Probe test on day 5.
**Negative symptoms**	*Dyadic social interaction*	↔	Free social interaction in novel cage with same-genotype interaction pairs

↑=increased, ↓=decreased, ↔=unaltered in *Df(h1q21)/+* mice compared to wildtype littermates.

AEP, auditory evoked potentials; AMPH, amphetamine; MEST, maximal electro shock threshold test; PCP, phencyclidine;PPI, prepulse inhibition; PTZ, pentylenetetrazole.

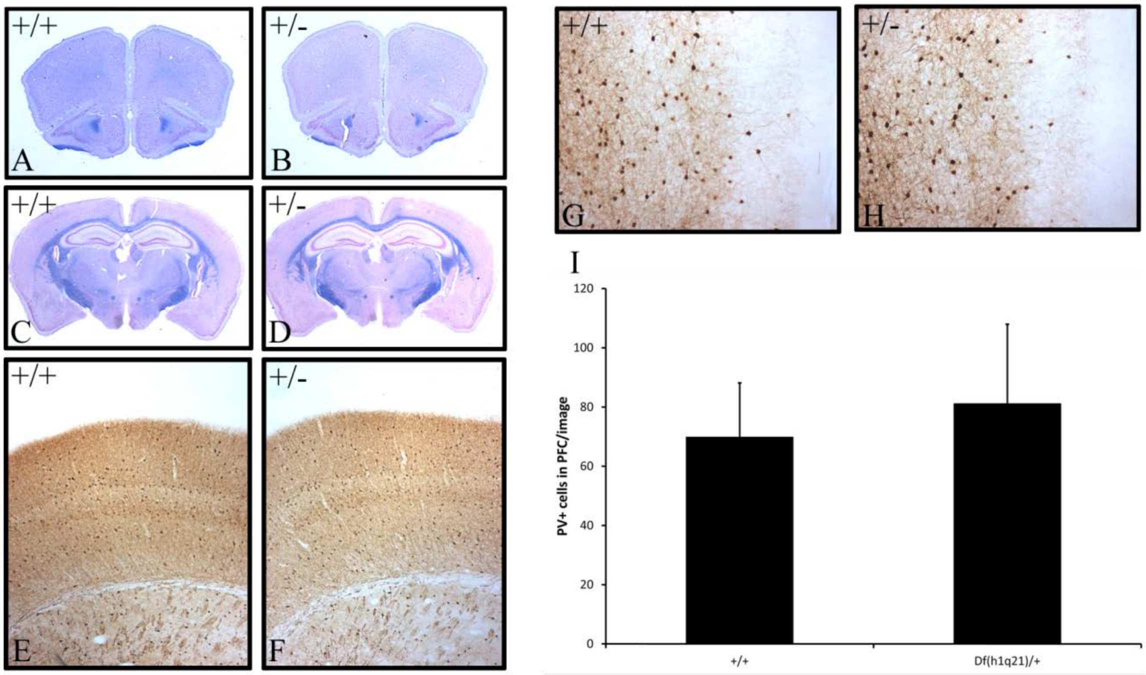

Figure S1. Histological evaluation of gross brain morphology. Solochrome staining was performed to compare brain morphology and myelination between genotypes (forebrain, A, B), (midbrain, C, D). Parvalbumin immunostaining was used to examine the number and distribution of parvalbumin in the cortex (E-H). Quantification of the number of PV cells in the prefrontal cortex is shown in the graph (I).

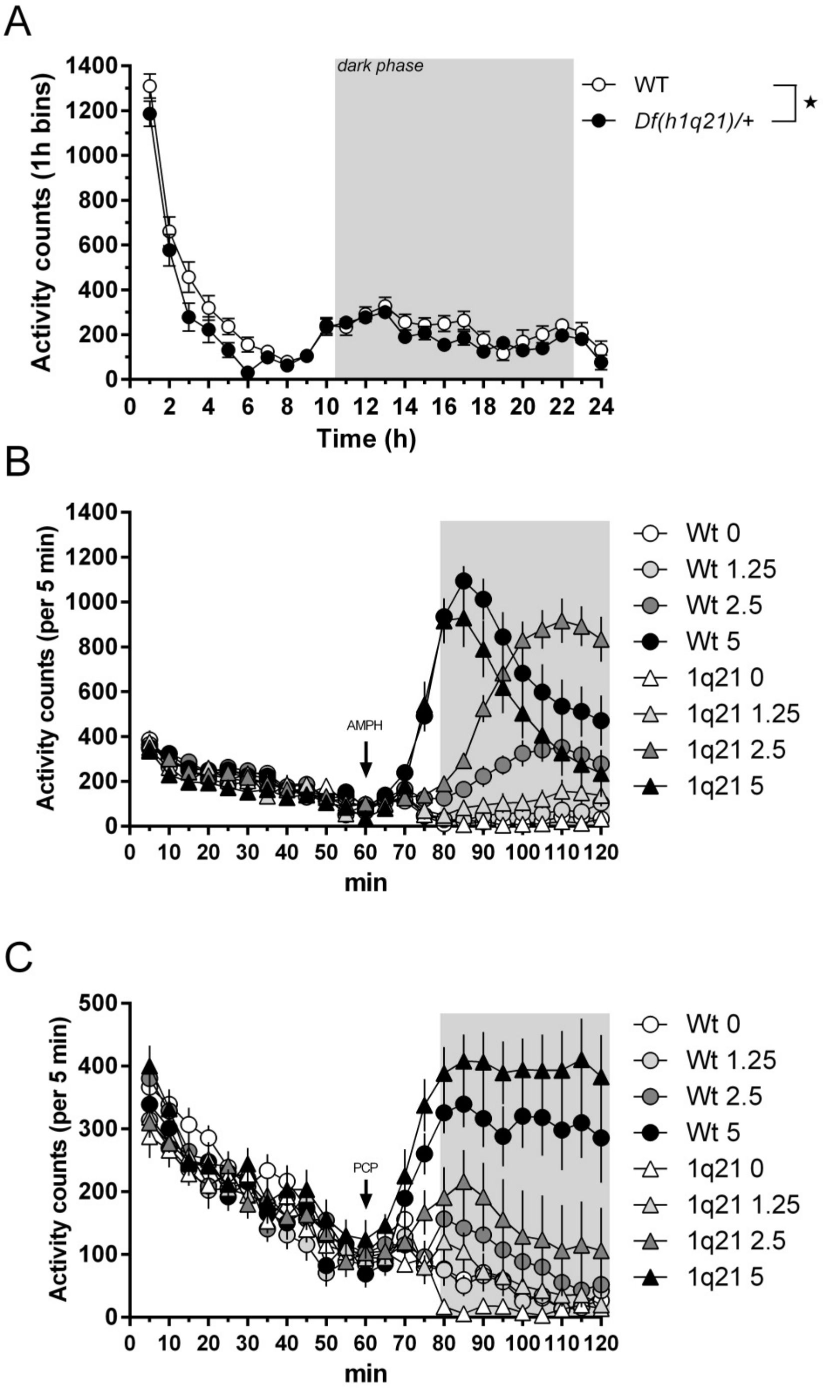

Figure S2. (**A**) Basal 24h locomotor activity in 1h min bins. Grey area symbolizes lights off (**B**) Horizontal activity in 5 min bins following amphetamine (AMPH) administration (0, 1.25, 2.5 and 5 mg/kg, s.c.) (**C**) Horizontal activity in 5 min bins following phencyclidine administration (PCP, 0, 1.25, 2.5 and 5 mg/kg, s.c.) Data presented as means +/-SEM. Grey area marks time period used for statistical analysis in Figure 2. ⋆ $p<0.05$, effect of genotype following mixed-model ANOVA.

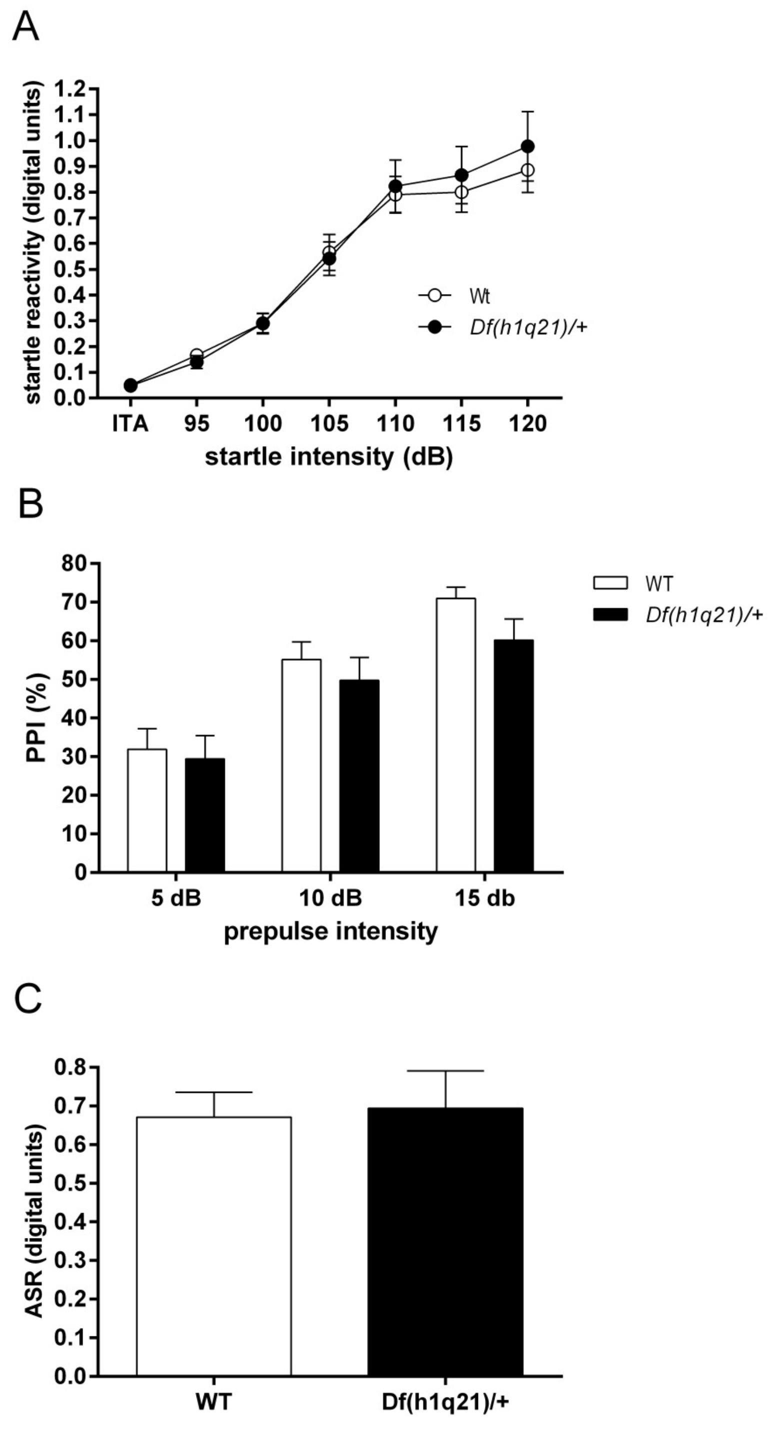

Figure S3 (**A**) Basal acoustic startle response (**B**) Prepulse inhibition (PPI) (**C**) Acoustic startle response (ASR) during PPI test. No significant differences were observed. Data presented as means +/-SEM.

.

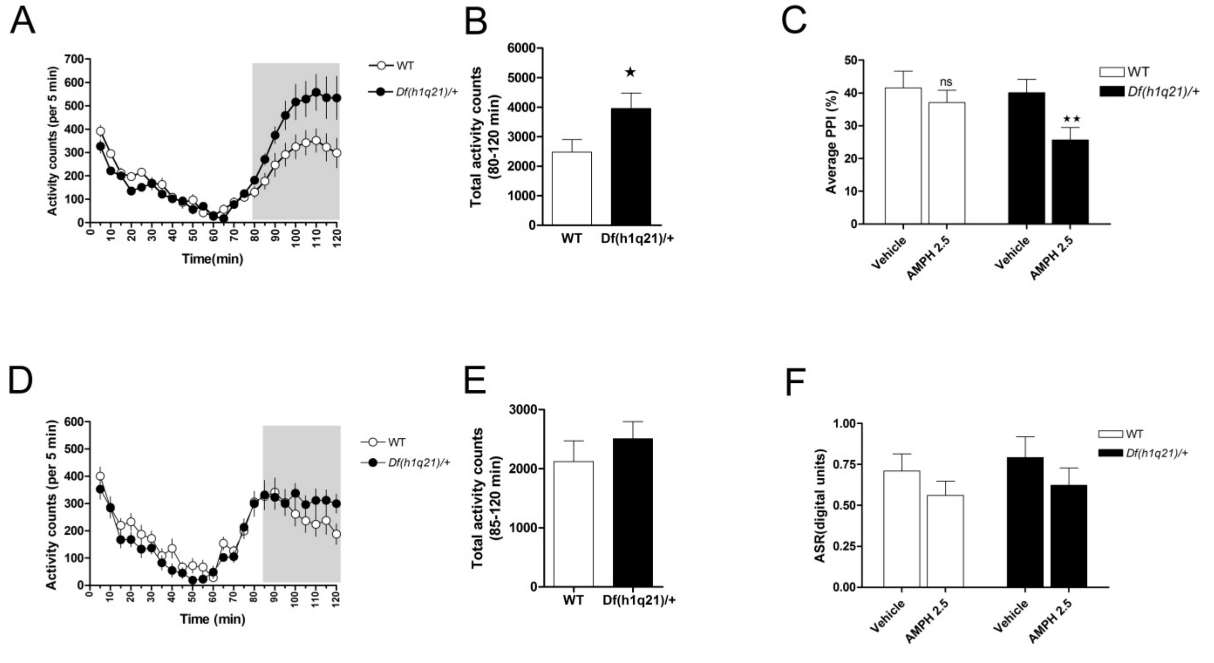

Figure S4. Replication of psychostimulant-induced changes in locomotor activity and prepulse inhibition (PPI). (**A**) Horizontal activity in 5 min bins following amphetamine (AMPH) administration (2.5 mg/kg, s.c.). (**B**) Horizontal activity summary for 80-120 bins in A. ⋆ $p<0.05$ following t-test. (**C**) PPI following AMPH administration (2.5 mg/kg). ⋆⋆ $p<0.01$ vs vehicle, Bonferroni corrected post-hoc test following mixed model ANOVA. (**D**) Horizontal activity in 5 min bins following phencyclidine (PCP) administration (5 mg/kg, s.c.). (**E**) Horizontal activity summary for 85-120 bins in D. (**F**) Average acoustic startle response (ASR) from PPI experiment in C. Data presented as means +/-SEM. Grey area in A and D marks time period used for statistical analysis in A and D.

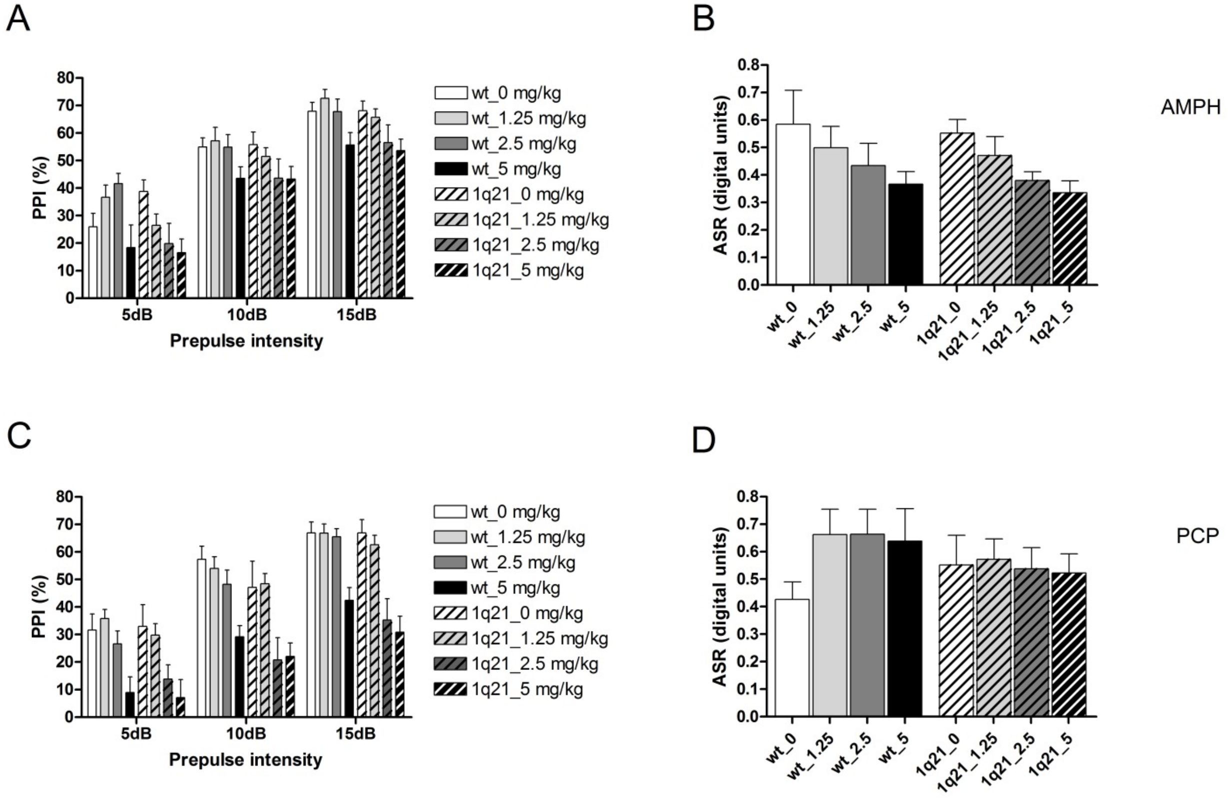

Figure S5. Psychostimulant-induced changes in prepulse inhibition (PPI) and acoustic startle response (ASR) *see Figure 2 C-D* (**A**) PPI for all prepulse intensities following amphetamine (AMPH) administration (0, 1.25, 2.5 and 5 mg/kg, s.c.) (**B**) ASR data from A (**C**) PPI for all prepulse intensities following phencyclidine (PCP) administration (0, 1.25, 2.5 and 5 mg/kg, s.c.) (**D**) ASR data from C. Data presented as means +/-SEM.

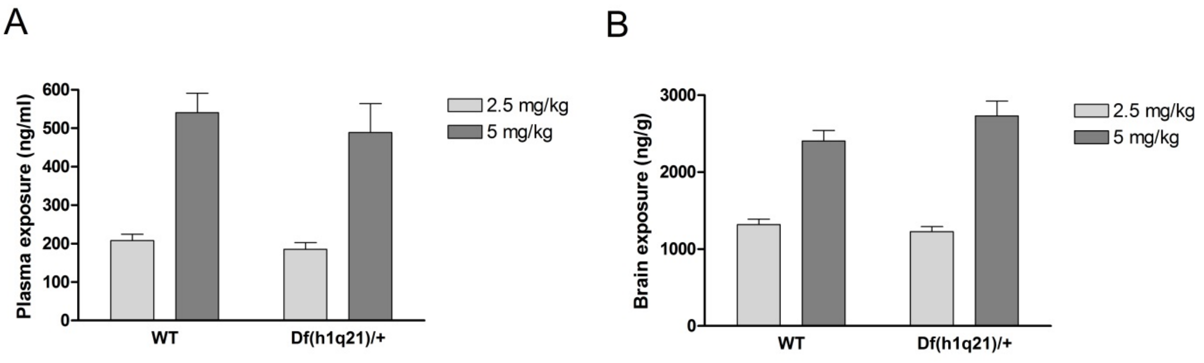

Figure S6. Exposure of amphetamine 1h after s.c. administration (**A**) Plasma exposure (**B**) Brain exposure. Data presented as means +/-SEM. n=6-8/group

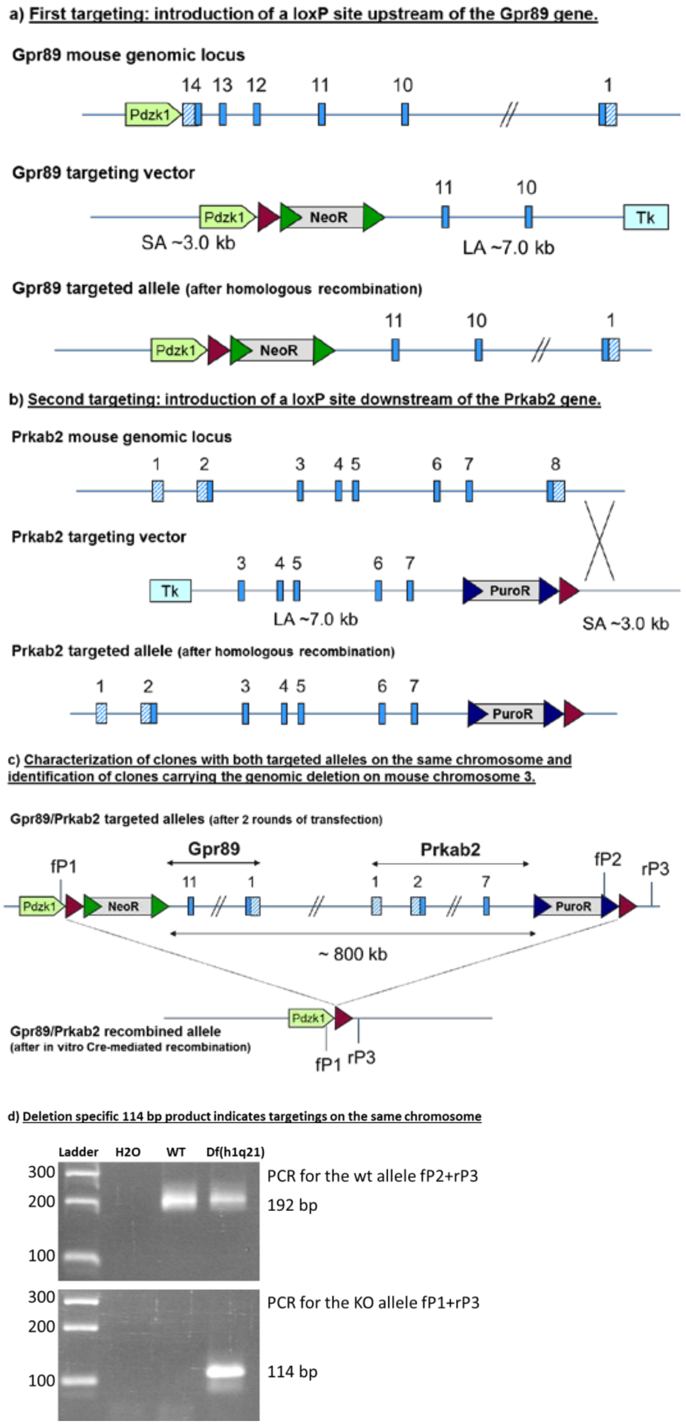

Figure S7 Generation of Df(h1q21) mice. A) WT Gpr89 loci and the corresponding targeted loci following introduction of a lox P site and Neomycin resistance gene. B) WT Prkab2 loci and the

corresponding targeted loci following introduction of a lox P site and Puromycin resistance gene. C) Cre-induced recombination between loxP sites in cis leads to around 800 kb deficiency, leaving only a single Lox P site. Primers for genotyping the wt and recombined (KO) allele are shown (forward primers fP1, fP2 and reverse primer rP3). D) Due to the recombination of the loxP sites, a diagnostic 114-bp PCR product can be detected in Df(h1q21) mice. The 192 bp PCR product is internal control to show that DNA is present in all samples. Diagrams are not drawn to scale.
